# Evaluation of Exome and Genome Sequencing for Critically Ill Pediatric Cardiac Patients

**DOI:** 10.21203/rs.3.rs-6314694/v1

**Published:** 2025-05-06

**Authors:** Angela C. Onorato, Rachel Gosselin, Bimal P. Chaudhari, Chance Alvarado, Peter White, Vidu Garg, Amee M. Bigelow

**Affiliations:** The Heart Center at Nationwide Children’s Hospital; The Steve and Cindy Rasmussen Institute for Genomic Medicine, The Abigail Wexner Research Institute, Nationwide Children’s Hospital; The Steve and Cindy Rasmussen Institute for Genomic Medicine, The Abigail Wexner Research Institute, Nationwide Children’s Hospital; The Heart Center at Nationwide Children’s Hospital; The Steve and Cindy Rasmussen Institute for Genomic Medicine, The Abigail Wexner Research Institute, Nationwide Children’s Hospital; The Heart Center at Nationwide Children’s Hospital; The Heart Center at Nationwide Children’s Hospital

**Keywords:** Congenital heart disease, Next generation sequencing, Genome sequencing, Exome sequencing, Cardiac intensive care unit

## Abstract

Genetic testing guidelines for children in cardiac intensive care units (CICUs) are lacking despite a high prevalence of genetic diseases among this population. Advances in next-generation sequencing (NGS) technologies, especially exome and genome sequencing (ES/GS), enable a more comprehensive genetic evaluation than more traditional testing modalities such as chromosomal microarray (CMA). While testing recommendations exist for cardiomyopathies, primary arrhythmias, and pulmonary hypertension (PH), broad application of NGS, especially ES/GS, across indications for admission to CICUs has not been recommended. We aimed to evaluate the diagnostic efficacy of ES/GS in critically ill pediatric patients with cardiac disease via a retrospective chart review of patients who underwent clinical ES/GS in a quaternary hospital’s pediatric CICU between January 2020 and August 2023. Forty-nine patients underwent ES/GS. Primary cardiac phenotypes included congenital heart disease, ventricular dysfunction, arrhythmia, and PH. Diagnostic results were found in 22 patients (44.9%) with 18/22 (81.8%) linked to cardiac phenotypes. Diagnostic yield was not different among primary cardiac phenotype groups but was higher in patients with ECA. CMA and gene panels would have failed to make a substantial proportion (80.6% and 36%, respectively) of diagnoses made by ES/GS. As NGS technologies and capabilities to interpret ES/GS data mature, diagnostic abilities in pediatric cardiac disease will continue to advance.

## Introduction

Pediatric cardiovascular disease encompasses a wide spectrum of conditions including congenital heart disease (CHD), primary arrhythmias, cardiomyopathies, and pulmonary hypertension (PH). Recognition of the underlying genetic etiology and the pivotal role genetics plays in the pathogenesis of pediatric cardiovascular disease is increasing. Recent literature suggests that pathologic genetic variation may explain at least 40% of CHD [1], 20% of primary arrhythmias [2,3], 20–40% of cardiomyopathies [2–5], and 3% of PH [6]. While guidelines exist for the use of next-generation sequencing (NGS) in the latter groups [4–7], there are currently no broad recommendations for NGS for CHD in the cardiac intensive care unit (CICU) [8,9]. Etiologic genetic factors have been found in both isolated CHD and CHD associated with extracardiac anomalies (ECA), highlighting the importance of understanding pathogenic variation for elucidating disease mechanisms, guiding therapy, assessing effects on post-operative and long-term outcomes, and determining familial recurrence risk [10–14].

Genetic testing is increasingly integrated into the diagnostic approach for pediatric heart disease. Chromosomal microarray analysis (CMA) is a first-line genetic test in patients with CHD [8,15]. CMA can detect aneuploidies and copy number variants (CNVs) involving smaller regions of DNA. While CMA offers a higher diagnostic yield than karyotyping, it cannot detect CNVs below approximately 5000 bp, small insertions or deletions (indels), or single nucleotide variants (SNVs) [16]. Consequently, CMA fails to diagnose a significant proportion of patients with heart disease with genetic etiologies [15–18], with diagnostic yields of 8–24% [16,18–21].

Advances in NGS technologies have revolutionized genetic testing, with the American College of Medical Genetics and Genomics (ACMG) recommending exome or genome sequencing (ES or GS) as first- or second-line genetic tests for congenital anomalies, including CHD [22]. However, while existing cardio-genetic testing guidelines recognize the potential value of ES or GS [4,5], they emphasize the use of disease-specific gene panels for conditions such as primary ventricular arrhythmias, cardiomyopathies, and PH [4–7]. Outside of these indications, most current recommendations in CHD do not include broad use of NGS technologies aside from occasional use of CHD gene panels in specific contexts [8,9]. Genomic sequencing using ES/GS is increasingly feasible, with high diagnostic yields in various pediatric populations, including critically ill infants, cardiac patients, and noncardiac patients [3,23–25]. We herein aimed to assess the diagnostic yield of ES and GS used in pediatric patients with a wide spectrum of cardiac disease presenting with critical illness at a single pediatric institution. We described the mutational spectrum of diagnostic variants and explored whether variants reported on ES/GS would likely have been reported on commercial panels or detected by CMA.

## Materials and methods

We conducted a retrospective review of all patients who underwent clinical ES or GS in the cardiothoracic intensive care unit at a single quaternary care children’s hospital from January 2020 to August 2023. Institutional Review Board approval was obtained from Nationwide Children’s Hospital, and informed patient consent was waived. Data collected included demographic information (age, sex, ethnicity, and race) and clinical characteristics including presence of extracardiac anomalies, cardiac phenotype, and clinical outcome. Race and ethnicity were reported as included in the medical record using hospital-based categories, which were based on patient/family self-report. Study data were collected and managed using Research Electronic Data Capture tools (REDCap) hosted at Nationwide Children’s Hospital. The data that support the findings of this study are available from the corresponding author upon reasonable request.

### Classification of cardiac and extracardiac phenotype

Patients were categorized into one of four primary cardiac phenotype groups: CHD, ventricular dysfunction, arrhythmia, and PH. In cases of overlapping cardiac phenotypes of CHD and arrhythmia, priority was given to CHD. Cases with both CHD and PH were placed in the PH group if the primary indication for genetic testing was PH. If ventricular dysfunction was the primary reason for genetic testing, the patient was included in the ventricular dysfunction group. Patients with both CHD and cardiomyopathy were assigned to the CHD group. One patient with cardiac rhabdomyomas was classified as “other”. Patients were further classified by extracardiac anomaly (ECA) status with patients classified as isolated heart disease if they had no identifiable ECA. Acquired multi-systemic diseases or systemic sequelae of CHD were not considered ECA.

### Genetic testing outcomes and variant level analysis

**Figure 1:**
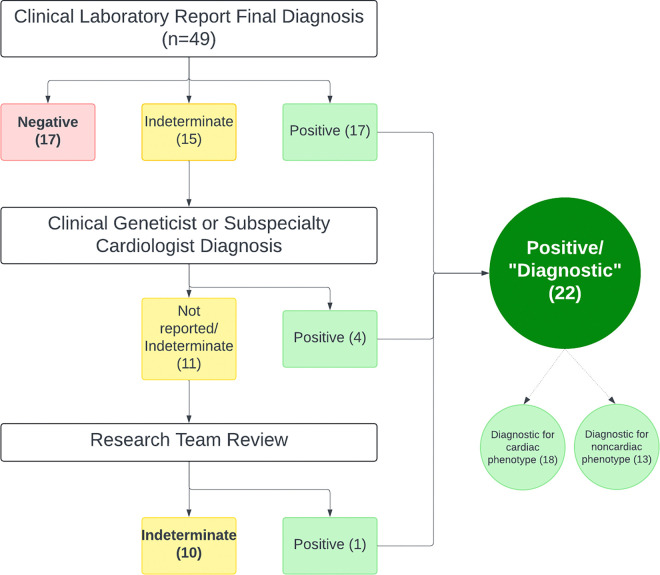
Flow chart describing the methodology used to classify ED and GS results. Tests designated as “positive” in clinical reports were classified as positive/”diagnostic” for the study. Additionally, results reported as indeterminate by the laboratory were reviewed by a subject matter expert (clinical geneticist or cardiology subspecialist with expertise in the phenotype or gene of interest) and, if considered diagnostic, they were classified as diagnostic for purposes of the study. Remaining indeterminate results underwent review by the research team (genetic counselor, genetic cardiologist, clinical geneticist, and cardiac intensivist). One patient was deemed to have a genotype-phenotype match and classified as diagnostic.

All available clinical genetic testing results were collected for all subjects, which included karyotype, CMA, specific variant tests, gene panels, and ES/GS. ES/GS test results were classified using modified Clinical Sequencing Evidence-Generating Research (CSER) Sequence Analysis and Diagnostic Yield (SADY) workgroup criteria [26,27] as outlined in Figure 1. Furthermore, diagnostic tests were adjudicated as “diagnostic for cardiac phenotype” and/or “diagnostic for noncardiac phenotype” to describe the phenotypic relationship. The research team, which included a cardiologist, clinical geneticist, genetic counselor, and cardiac intensivist, reviewed testing results. Diagnostic variants were analyzed based on AMP/ACMG classification in the testing report. Variants were assessed by the research team to determine whether karyotype or CMA were likely to detect each variant. Based on gene and variant location and type, the research team determined whether each variant would be detectable on commercially available gene panels for cardiac pathologies at four laboratories: Invitae, GeneDx, Mayo Clinic, and Prevention Genetics. The following test IDs were reviewed (Invitae 02101, 02211, 02213, 02214, 02201, 02212, 02261, 02262, 04151, 02251, 02263, 04204, 02351, 02301; GeneDx 695, 727, 883, 935, J552, 481, J555, 919, T998, TA06, TJ07; Mayo Clinic CACMG, CARGG, CCMGG, ARVGG, CPVTG, DCLNG, HCMGG, LQTSG, NSRGG, SQTSG, SCN5A [accessed September 26, 2024], Prevention Genetics 7739, 8983, 5251, 3057, 10363, 10323, 10423, 10325, 10231, 10329, 10261, 2609, 2663, 1339, 5263, 1313, 1333, 10249, 1773, 2663, 13008, 10229, 13097, 10327, 10071, 10405, 8819, 8475, 10163, 10363, 15737, 12625 [accessed October 24, 2024]).

### Statistical analysis

Patient demographic and clinical characteristics are described using median with interquartile range for continuous variables and frequency (percent of total) for categorical variables. Wilcoxon rank sum tests were used to compare age at genetic testing by primary cardiac phenotype and presence of extracardiac anomalies. ECA and diagnostic yield were compared between cardiac phenotype subgroups using Fisher’s exact test due to the relatively small sample sizes and the categorical nature of the data. P-values were not adjusted for multiple comparisons. All statistical analyses were performed using R version 4.2.2 (R Core Team, Vienna, Austria).

## Results

**Figure 2:**
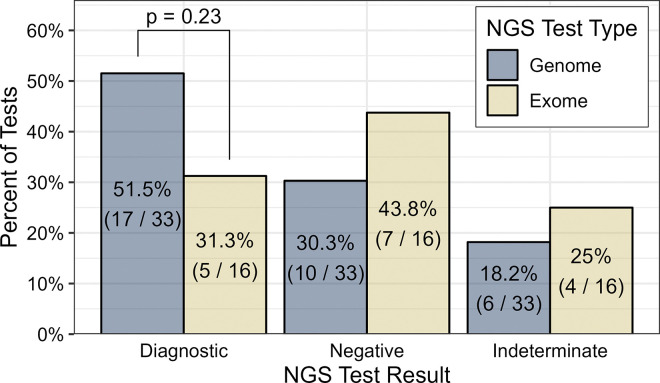
Rates of diagnostic, negative, and indeterminate results by NGS test type (exome or genome). The odds of a diagnostic test in the GS group are 2.34 times those in the ES group, however this was not statistically significant (Fisher exact test p = 0.23).

A total of 49 patients (53.1% female, 70.8% white) underwent clinical ES/GS during the study period. Median age at time of testing was 46 days (IQR 7–609). Patient characteristics are summarized in Table 1. Half of the patients had CHD (25/49, 51.0%), and 26/49 (53.1%) had ECA. GS was the predominant molecular test (33/49, 67.3%) with a 51.5% diagnostic rate (17/33) compared to ES (5/16, 31.3%), as shown in Figure 2.

Overall, ES/GS yielded diagnostic results for 22/49 (44.9%) patients, while 17/49 (34.7%) were negative, and 10/49 (20.4%) were indeterminate by Clinical Sequencing Evidence-Generating Research (CSER) Sequence Analysis and Diagnostic Yield (SADY) criteria. Among diagnostic results, 18/22 (81.8%) were diagnostic for the cardiac phenotype of the patient tested, yielding a diagnostic rate of 36.7% for cardiac phenotypes. The other 4 diagnostic tests were diagnostic only for non-cardiac phenotypes. Online Reference Table 1 lists case-level data for all diagnostic cases.

Thirty-one unique variants were identified among the 22 diagnostic tests and are summarized in Table 2. Most of these variants (83.9%) were pathogenic/likely pathogenic, with 16.1% variants of uncertain significance. Notably, 25/31 variants (80.6%) were SNVs or indels, and thus would have been undetectable on chromosomal microarray. Among the 25 variants associated with the 18 tests diagnostic for cardiac phenotypes, 9/25 (36.0%) would not have been detected on widely used gene panels for cardiac diseases (Invitae, Prevention Genetics, GeneDx, and Mayo Clinic Laboratories) because they were either CNV or SNVs in genes not included on these panels. Table 3 lists the genes associated with results diagnostic for cardiac disease, stratified by phenotype. Online Reference Table 2 lists detailed variant information for all diagnostic cases.

**Figure 3:**
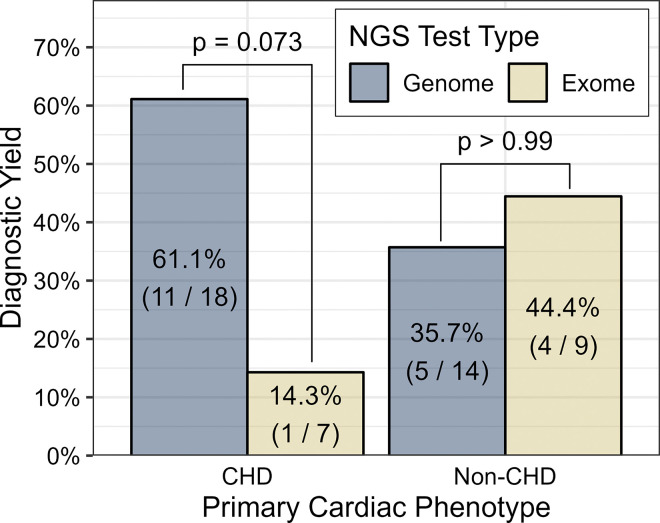
Rates of diagnostic results by NGS test type and cardiac phenotype group (CHD vs. non-CHD).

Patients with CHD (n=25) were compared to a combined group of patients with either ventricular dysfunction, arrhythmia, or PH (n=23; hereafter referred to as the non-CHD group), due to the disparity in recommendations for NGS use between these populations. The results of this analysis are shown in Table 4. The CHD group was significantly younger at the time of genetic testing than the non-CHD group (p = 0.005). There was a significantly higher prevalence of ECA in the CHD group compared to the non-CHD group (72.0% vs. 30.4%) (p = 0.009). However, the diagnostic yield did not differ significantly between the CHD and non-CHD groups. GS demonstrated a higher diagnostic yield (61.1%) for the CHD group than ES (14.3%), though this did not meet statistical significance (p=0.073) (Fig. 3).

**Figure 4:**
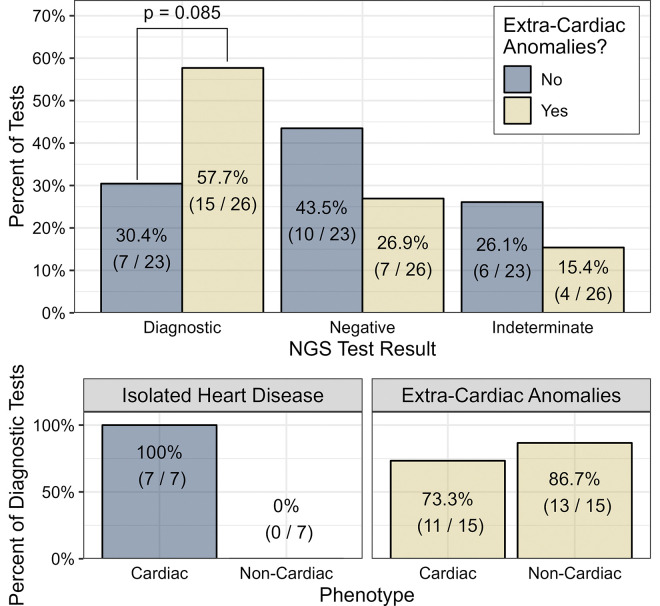
Rates of diagnostic, negative, and indeterminate results by the presence of extracardiac anomalies. The lower subplots depict whether the patients’ cardiac or noncardiac (or both) phenotypes are explained by diagnostic tests.

Additional analyses were performed on patients stratified by ECA status. Patients with ECA predominantly had CHD (18/26, 69.2%) and were significantly younger than those with isolated heart disease (median age 26 days [IQR 4.5–71] vs. 905 days [IQR 11.5–4692]) (p=0.014). The diagnostic rate of ES/GS for the ECA group trended higher than the isolated heart disease group (57.7% vs. 30.4%) (p=0.085) (Fig. 4). Of the 15 diagnostic tests in the ECA group, 10 (66.7%) were diagnostic for cardiac phenotypes and 13 (86.7%) were diagnostic for non-cardiac phenotypes.

## Discussion

Our retrospective review of ES and GS in the pediatric CICU revealed several key findings. The diagnostic yield of ES/GS in our cohort was 44.9%, with no significant difference between CHD and non-CHD patients. The diagnostic yield was particularly high in patients with extracardiac anomalies. Furthermore, many diagnostic tests in our cohort would not have been identified with chromosomal microarrays (80.6%) or common cardiac gene panels (36.0%) alone. GS, which accounted for two-thirds of NGS testing, yielded a diagnosis for over half of patients, compared to a diagnostic yield of less than one-third for ES. Our results underscore the potential impact of ES/GS in the critically ill pediatric cardiology population.

The overall diagnostic yield of ES/GS in our study (22/49, 44.9%) is consistent with previous studies of ES/GS in pediatric cardiology, which reported diagnostic yields ranging from 27–46% [3,25,28]. However, unlike prior studies focused exclusively on cardiac genetic etiologies, our yield includes both cardiac and noncardiac diagnoses, which contributed to our slightly higher overall diagnostic yield. When accounting only for cardiac phenotypes, the diagnostic yield was lower (36.7%), albeit still in line with prior studies [3,25,28]. Understanding both cardiac and non-cardiac diagnoses is crucial, as either may impact complications, prognosis, and outcomes in critically ill patients. This study highlights the high diagnostic yield of ES/GS across a broad spectrum of pediatric cardiac phenotypes, as the yield was comparable between the CHD group and the combined group of arrhythmia, ventricular dysfunction, and PH patients. The genetic contributions to ventricular dysfunction, primary arrhythmias, and PH are better understood, largely because these conditions are more prevalent in adults and have been extensively studied. In contrast, CHD has not received the same level of focus, resulting in fewer established guidelines for genetic evaluation. Current recommendations for NGS are typically limited to conditions such as cardiomyopathies, arrhythmias, and PH, often involving targeted gene panels [4–7]. A recent ACMG statement suggested ES or GS could be used as a first- or second-tier diagnostic test for any congenital anomalies, including CHD [22]. However, ES/GS is not consistently applied in clinical practice and most CHD guidelines continue to recommend chromosomal microarray as a first-line diagnostic test for certain anatomic lesions [8,9,15].

Our study indicates that ES/GS can be as valuable in CHD as in other pediatric cardiac phenotypes. It is important to note, however, that the CHD cohort in this study had a higher prevalence of ECA (72.0%) compared to the previously reported estimate of ECA in CHD of 22.3% [29], likely because ES/GS were being sent more frequently in the ECA population than the isolated CHD population in our institution during the study period. This may have inflated the diagnostic yield in this population, as it is well known that patients with ECA are more likely to carry a genetic diagnosis [3,17,30–34]. In our study, patients with ECA were nearly twice as likely to have a diagnostic result compared to those with isolated cardiac disease, which is consistent with prior studies [29–33]. However, about one-third of patients with isolated cardiac disease also had a diagnostic ES/GS result, highlighting the potential impact of broad genetic testing in isolated cardiac disease rather than targeting only patients with ECA. This aligns with recent literature showing that the presence of ECA has a low-moderate screening performance as an indication for ES/GS and that performing ES/GS based on ECA status leads to underdiagnosis in patients with isolated CHD [34].

This study demonstrated a higher rate of detection of pathogenic genetic variation using GS than ES and using ES/GS compared to traditional genetic tests, such as karyotype and chromosomal microarray. While the study was likely underpowered to detect a statistically significant difference between GS and ES, GS had a diagnostic yield of 51.5% compared to 31.3% for ES. Recent literature also supports that GS has higher diagnostic yield than ES [3,35]. Historically, karyotyping and CMA have been more broadly recommended for pediatric cardiac diseases [8,9,15], however, our results suggest that these methods alone would miss a substantial proportion of underlying genetic diagnoses. Additionally, the genomic variation they can detect could largely be detected by GS (except possibly for some structural variation). Gene panels can detect SNVs and indels, and thus detect a range of pathogenic variants distinct from karyotype or CMA [17]. However, common current cardiology-focused gene panels alone would have missed over one-third of the variants detected in our study, which aligns with recent literature indicating higher diagnostic yield in ES/GS compared to gene panels [21,28,36]. In an era of rapid genomic advancement, gene panels do not capture emerging diagnostic findings and do not facilitate the future evaluation or re-interpretation of newly identified clinically relevant genes. The genes included in specific gene panels vary by laboratory [37], the addition of new genes to panels often lags behind evidence-based data [36], and the chances of finding VUS may actually be higher on multi-gene panels than ES/GS [38]. Additionally, while the cost of ES or GS testing is generally higher than gene panels, the overall cost benefit of a positive diagnostic result favors the more comprehensive test, especially in critically ill patients [38–40].

Limitations of this study included its retrospective nature, small sample size, and potential selection bias due to the nature of ES/GS performed during this study period. The effects of genetic diagnoses on outcomes were outside the scope of this study. The study’s confinement to a single center and specialized CICU also limits generalizability of the findings to the broader pediatric cardiology population, though this is an area for future investigation. The small sample size prohibited an analysis of more specific cardiac substrates, thus was limited to classes of pathologies. Within each class, there is likely variability to the diagnostic yield of genetic testing, but this was unable to be reported in this study.

In summary, our study demonstrates a high diagnostic yield of ES/GS in the pediatric CICU setting, especially in patients with extracardiac anomalies. Moreover, both GS and ES demonstrate significant clinical value compared to targeted gene panels or CMA. The high diagnostic yield and ability of ES/GS to uncover the genetic etiology of a patient’s disease or provide evidence for the discovery of novel genetic mechanisms make them invaluable. These findings support the broader implementation of ES/GS for critically ill pediatric cardiac patients to improve diagnostic outcomes. Future studies involving larger, multi-center cohorts are warranted to further validate these findings and assess the broader applicability of ES/GS in pediatric cardiology.

## Figures and Tables

**Table 1.** Patient characteristics

Characteristic	N = 49^a^
**Age at genetic testing (days)**	46 (7–609)
≤1 year	36 (73.5%)
>1 year	13 (26.5%)
**Sex**	
Male	23 (46.9%)
Female	26 (53.1%)
**Race**	
White	34 (70.8%)
Black/African American	9 (18.8%)
Other race/multiple races	5 (10.4%)
Unknown	1
**Deceased**	17 (34.7%)
Age at Death (Days)	149 (41–243)
**Cardiac Phenotype**	
Congenital heart disease	25 (51.0%)
Non-congenital heart disease	23 (46.9.0%)
Ventricular Dysfunction	13 (56.5%)
Arrhythmia	6 (26.0%)
Pulmonary hypertension	4 (17.4%)
Other (rhabdomyoma)	1 (2.0%)
**Extracardiac Anomaly Present**	26 (53.1%)
**Genetic Testing Performed**	
Karyotype	16 (32.7%)
Chromosomal microarray	23 (46.9%)
Gene panel	11 (22.4%)
Exome sequencing	16 (32.7%)
Genome sequencing	33 (67.5%)

a Median (IQR); n (%)

**Table 2.** Analysis of variants detected by ES/GS on diagnostic tests

Variant Description	N(%)
**Variant Types**	
SNV	21 (67.7%)
Indel	4 (12.9%)
CNV (all from GS)	6 (19.4%)
**Variant Classification**	
Pathogenic	12 (38.7%)
Likely pathogenic	14 (45.2%)
Variant of uncertain significance	5 (16.1%)
**Detection of Diagnostic Variants on Other Modalities**	
Variant not detectable by karyotype/CMA (n=31)	25 (80.6%)
Variant not detectable by cardiac gene panels (n=25)^a^	9 (36.0%)

a Includes cardiac gene panels from Invitae, GeneDx, Mayo Clinic, and Prevention Genetics Laboratories

**Table 3.** Identified genes grouped according to the cardiac phenotype of patients that had diagnostic tests for their cardiac phenotypes.

Cardiac Phenotype	Specific Genes/Loci Implicated				

Congenital heart disease	22q11.21 deletion	FKTN	KMT2D	LZTR1	TXNL4
	47, XX, +21	FLNA	LRRC56	PTPN11	VEGFA

Ventricular dysfunction	ACTC1	PPCS	RREB1	TAZ	

Arrhythmia/cardiac arrest	CASQ2	KCNH2	RYR2		

**Table 4.** Characteristics and ES/GS results by primary cardiac phenotype group

Characteristic	CHD, N = 25^a^	Non-CHD N = 23^a^	Difference^b^	p-value^c^
**Age at genetic testing (days)**	17 (7–60)	905 (11–4714)	−888	0.005
≤1 year	24 (96.0%)	11 (47.8%)	48.2	
>1 year	1 (4.0%)	12 (52.2%)	−48.2	
**Living**	13 (52.0%)	18 (78.3%)	−26.3	-
**Extracardiac anomalies**	18 (72.0%)	7 (30.4%)	41.6	0.009
**ES/GS Result**				
Diagnostic	12 (48.0%)	9 (39.1%)	8.9	0.57
Negative	9 (36.0%)	8 (34.8%)	1.2	
Indeterminate	4 (16.0%)	6 (26.1%)	−10.1	

a Median (IQR); n (%)

b Difference in median; difference in observed percent

c Wilcoxon rank sum test; Fisher’s exact test

## Abbreviations

CHD: congenital heart disease
PH: pulmonary hypertension
NGS: next-generation sequencing
CICU: cardiac intensive care unit
ECA: extracardiac anomalies
CMA: chromosomal microarray
CNV: copy number variant
Indel: small insertion or deletion
SNV: single nucleotide variant
ACMG: American College of Medical Genetics and Genomics
ES: Exome Sequencing
GS: Genome Sequencing
IQR: Inter-quartile range
VUS: variant of uncertain significance
